# Sarcomatous Change of Cerebellopontine Angle Ependymoma Following Radiosurgery: A Case Report

**DOI:** 10.7759/cureus.20864

**Published:** 2022-01-01

**Authors:** Nicholas B Dadario, Rachel Pruitt, Justin W Silverstein, Avraham Zlochower, Sewit Teckie, Manju Harshan, Randy S D'Amico

**Affiliations:** 1 Neurological Surgery, Lenox Hill Hospital/Donald and Barbara Zucker School of Medicine at Hofstra, New York, USA; 2 Neurology, Lenox Hill Hospital/Donald and Barbara Zucker School of Medicine at Hofstra, New York, USA; 3 Neurology, Neuro Protective Solutions, New York, USA; 4 Radiology, Lenox Hill Hospital/Donald and Barbara Zucker School of Medicine at Hofstra, New York, USA; 5 Radiation Oncology, Lenox Hill Hospital/Donald and Barbara Zucker School of Medicine at Hofstra, New York, USA; 6 Pathology, Lenox Hill Hospital/Donald and Barbara Zucker School of Medicine at Hofstra, New York, USA

**Keywords:** glioma, sarcoma, ependymoma, gliosarcoma, cerebellopontine angle, ependymosarcoma

## Abstract

Sarcomatous change in ependymal tumors is rare and has been poorly described. We report on a cerebellopontine angle lesion that demonstrated rapid progression two years after radiosurgery in a 73-year-old female patient. Histopathological diagnosis at clinical progression showed an ependymoma with sarcomatous change (“ependymosarcoma”) that was believed to be due to radiation. The patient underwent a complex tumor resection without complications using an exhaustive multi-modal neuromonitoring paradigm throughout the dissection and resection of the tumor. Given the limited available data on these rare tumors, we review their presentation, imaging, and histopathology in the context of the previous literature, and also discuss the management of these lesions in the cerebellopontine angle.

## Introduction

Gliosarcomas are rare central nervous system malignancies, and they account for approximately 2% of all glioblastomas (GBM) [1,2]. The treatment regimens for the condition are similar to those used for GBM, with a median survival rate of less than two years [1,3].

Uniquely, gliosarcomas display a characteristic dimorphic tumor pattern of glial and mesenchymal elements. The glial component is most commonly malignant astrocytoma [4], while the sarcomatous components can variably include fibroblastic, cartilaginous, osseous, smooth muscle, or adipose cell origins [5]. However, outside of the World Health Organization (WHO) classification of gliosarcomas as a variant of high-grade astrocytomas, sarcomatous changes also frequently occur in oligodendroglial tumors [6], and recently these changes have been described in ependymomas [4,7] and subependymomas [8].

While cytogenic studies have investigated the many possible molecular mechanisms of sarcomatous transformation [4], isolated reports have suggested the possible role of radiation therapy in inducing sarcomatoid changes in glial tumors, specifically anaplastic ependymomas [4,7,9,10]. The identification of these “ependymosarcomas” is critical given their tendency to display variable clinical behavior in a variety of cerebral locations, which is likely associated with a more aggressive phenotype [4]. However, more information is required to further understand their effective clinical management in a variety of cerebral locations as we continue to expand our understanding of their histopathological origins.

We report on a cerebellopontine angle lesion that demonstrated rapid progression two years after radiosurgery in a 73-year-old female patient. Histopathological diagnosis at clinical progression demonstrated an ependymoma with sarcomatous change believed to be due to radiation. We review the presentation, imaging, and histopathology in the context of previously published data on this rare condition.

## Case presentation

A 73-year-old female with a past medical history of hypertension, hyperlipidemia, and chronic obstructive pulmonary disease (COPD) presented to the hospital with progressive dizziness and gait instability, dysphagia, worsened bilateral hearing loss, and mild intermittent headache. The patient reported a previous syncopal episode and left-sided hearing loss three years prior, followed by tinnitus in the form of a “swish” sound. Workup in a local emergency department had demonstrated a previously treated 3.2 x 2.7 x 3.2-cm “salt and pepper” left cerebellopontine angle lesion arising from the medial aspect of the jugular bulb with compression of the brainstem and cerebellum, as well as fourth ventricle compression. Differential diagnoses had favored paraganglioma and included ependymoma and meningioma. Schwannoma and metastatic disease had been deemed less likely, and no abnormal nodularity or enhancement of the cranial nerve VII/VIII complexes had been seen. Bloodwork had shown no secretion of hormones. The patient had been recommended for stereotactic radiosurgery given her minimal symptoms at the time and her desire to avoid surgical intervention. She had elected to undergo linear-accelerator-based radiosurgery three years ago at an outside institution. She had been subsequently treated with fractionated stereotactic radiosurgery using 6 megavoltage photons to a prescription dose of 2500 cGy in five fractions over the course of seven days. She had been followed up with serial imaging for one year and then lost to follow-up.

**Figure 1:**
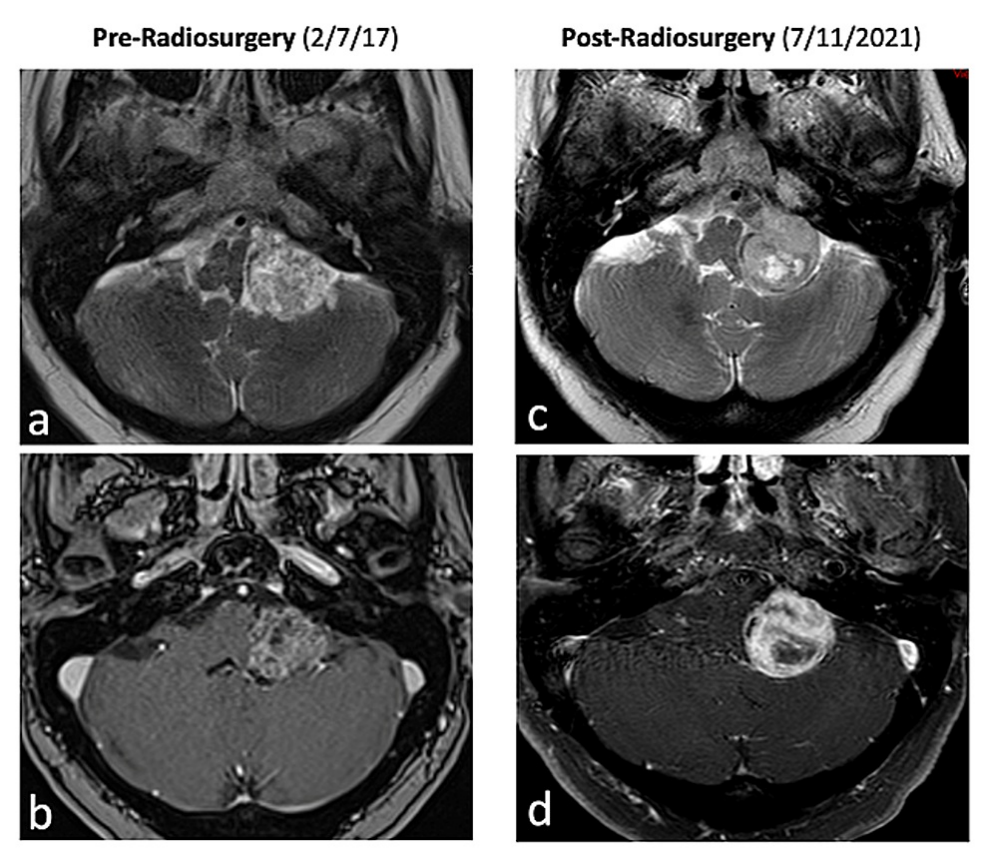
Original MRI brain imaging before and after radiation treatment Original brain imaging before (a-b) and after (c-d) radiation treatment is shown. (a) Axial T2-weighted image demonstrates a posterior fossa mass centered in the left cerebellopontine angle cistern and lateral medullary cistern with heterogenous T2 hyperintense signal. (b) Axial T1 post-contrast weighted image demonstrates heterogeneous enhancement. (c) Axial T2-weighted image demonstrates mild interval growth of the mass that shows an area of central T2 hyperintense signal due to cystic/necrotic changes. (d) Axial T1 post-contrast image demonstrates more prominent solid peripheral enhancement with central non-enhancement due to cystic/necrotic changes MRI: magnetic resonance imaging

**Figure 2:**
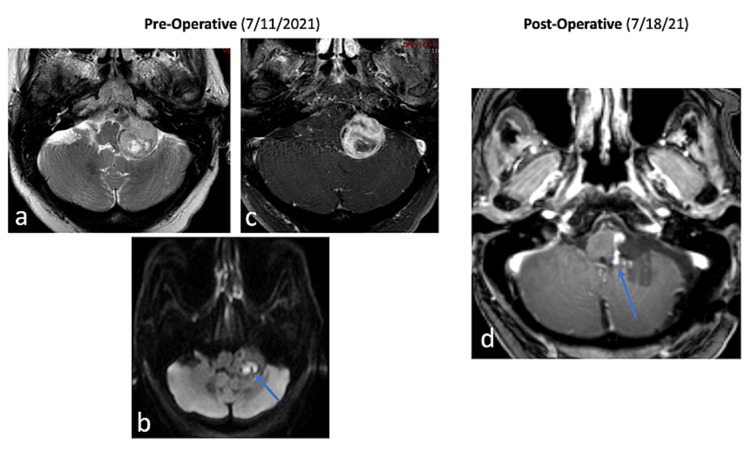
Pre- and postoperative imaging Preoperative images are shown in panels a-c and postoperative images in panel d. (a) Axial T2-weighted image demonstrates heterogeneous mass with an area of central T2 hyperintense signal due to cystic/necrotic changes. (b) Axial diffusion-weighted imaging (DWI) demonstrates restricted diffusion within the tumor (blue arrow on DWI). (c) Axial T1-weighted post-contrast image demonstrates prominent solid peripheral enhancement with central non-enhancement due to cystic/necrotic changes. (d) Postoperative MRI axial T1-weighted post-contrast image demonstrates resection of the mass with small residual nodular enhancing tumor along the medial margin (arrow) MRI: magnetic resonance imaging

On this admission, contrast-enhanced MRI of the brain on admission demonstrated progressive growth of the known lesion with a change in its radiographic characteristics (Figure 1). On physical examination, the patient was noted to have complete left hearing loss. Endoscopic evaluation of the vocal cords demonstrated complete vocal cord dysfunction on the left. Barium swallow also demonstrated swallowing dysfunction. Due to the progressive growth of the lesion and change in radiographic features despite previous radiosurgery, coupled with clinical worsening, the decision was made to proceed with surgical decompression and biopsy (Figure 2). The patient underwent a left retrosigmoid craniotomy and near-total resection of the lesion with residual left along the lateral brainstem. Intraoperative neurophysiological monitoring was completed to identify cranial nerves V-XII and is described below.

Neuromonitoring

A multi-modal neuromonitoring paradigm included somatosensory evoked potentials (SSEP), transcranial motor evoked potentials with limb recordings (TCMEP-L), corticobulbar motor evoked potentials (co-bulb MEP), brainstem auditory evoked responses (BAER), spontaneous cranial nerve electromyography (s-CNEMG), and evoked CN EMG (e-CNEMG).

SSEP and TCMEP-L were conducted in the standard fashion and were obtained prior to the incision and did not deviate from their established baseline. BAEPs were unreliable from the start due to the underlying binaural hearing loss. Co-bulb MEPs were only obtained from the trapezius muscles. All other muscles revealed compound muscle action potentials (CMAP) with single pulse stimulation, indicating that they were not being generated centrally but instead peripherally due to stimulus current spread.

Cranial Nerve EMG (Spontaneous and Evoked)

**Figure 3:**
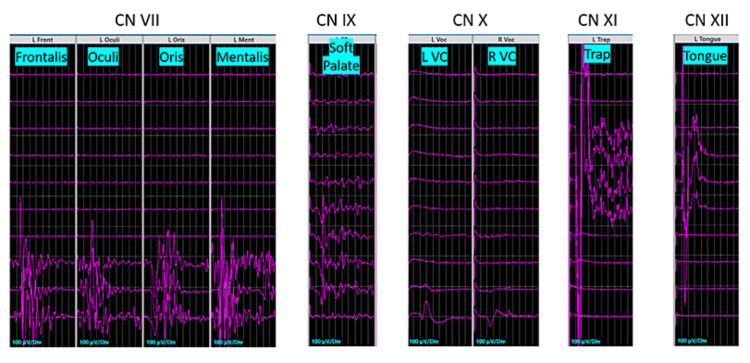
Identification of cranial nerves with dynamic stimulation Using an electrified monopolar ring dissector, we conducted dynamic stimulation throughout the course of the dissection and resection of the tumor. Using stimulus intensities between 0.05 mA and 0.5 mA, we were able to identify all motor cranial nerves targeted excluding CN V

**Figure 4:**
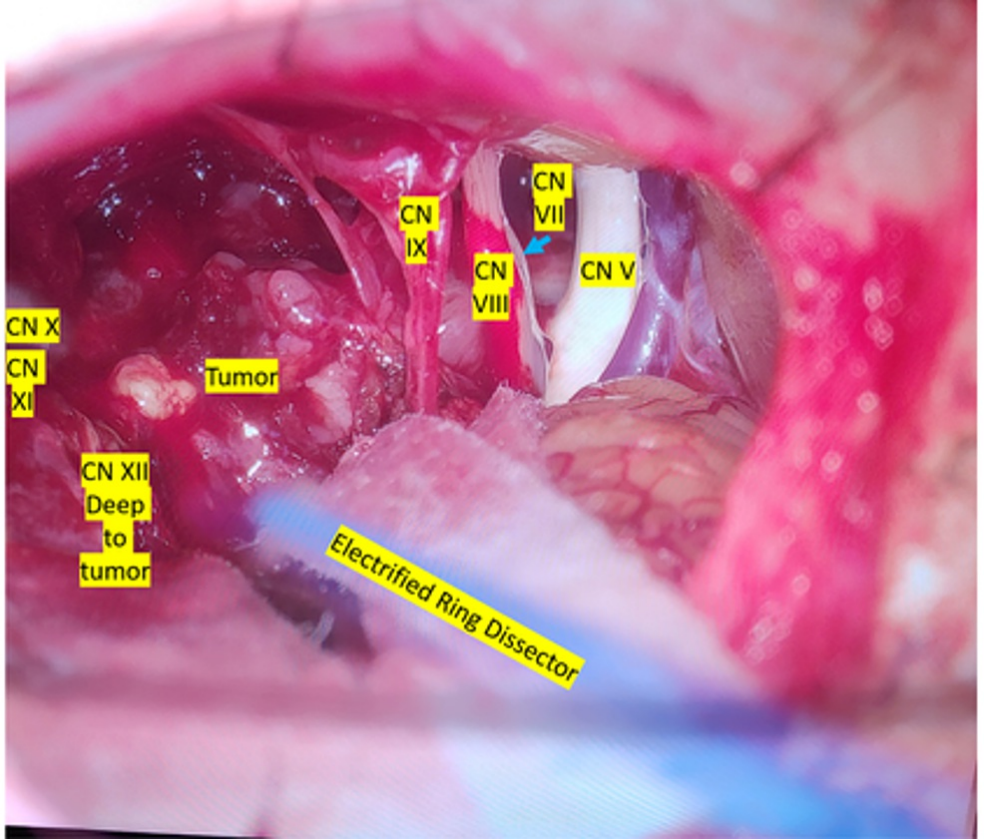
Anatomic intraoperative visualization of cranial nerves CN X was only confirmed with stimulation and not visually. The current figure demonstrates the area where we obtained an isolated response from CN X

Cranial nerves V, VII, IX, X, XI, and XII were targeted for CNEMG. Using an electrified monopolar ring dissector, we conducted dynamic stimulation throughout the dissection and resection of the tumor. Using stimulus intensities between 0.05 mA and 0.5 mA, we were able to identify all motor cranial nerves targeted excluding CN V, which was visualized instead (Figure 3). We were able to visualize all targeted CNs as well, excluding CN X, which we found electrically but never visually (Figure 4). We were also never able to activate CN IX proximally, though we were able to expose it and track it back to the brainstem. CN IX did stimulate electrically distally at the jugular foramen at 0.5 mA but never demonstrated proximal activation suggesting no obtainable response of CN IX at the brainstem.

Postoperative results

Tumor resection proceeded without complication. Postoperatively, the patient developed worsening dysphasia and delayed gastric emptying requiring a percutaneous endoscopic gastrostomy due to decreased calorie intake. She was able to swallow and receive oral intake consistent with her preoperative baseline neurologic status. The patient also underwent laryngoplasty with a return of normal vocalization. She was eventually discharged to acute rehabilitation. Pathology was consistent with a WHO grade II ependymoma with a high-grade spindle cell neoplasm component with a TERT (C228T) promoter mutation. Postoperative MRI demonstrated an STR with an expected residual tumor located along the vertebral artery/brainstem interface (Figure 2d). There was mild diffusion restriction within the lateral cerebellar hemisphere. Of note, a follow-up MRI one month postoperatively showed subtle progression of the disease at the area of residual tumor. Two months postoperatively, CT with contrast demonstrated continued progression. A few days later, the patient was found ill and down at home. CT demonstrated new subarachnoid and interlesional hemorrhage with dilation of the lateral and third ventricles with intraventricular hemorrhage. At the time of disease progression, she was made DNR/DNI; due to her continued decline, made worse by the hemorrhage, the decision was made to implement comfort measures, and she was later discharged to inpatient hospice where she subsequently passed away.

Pathology

**Figure 5:**
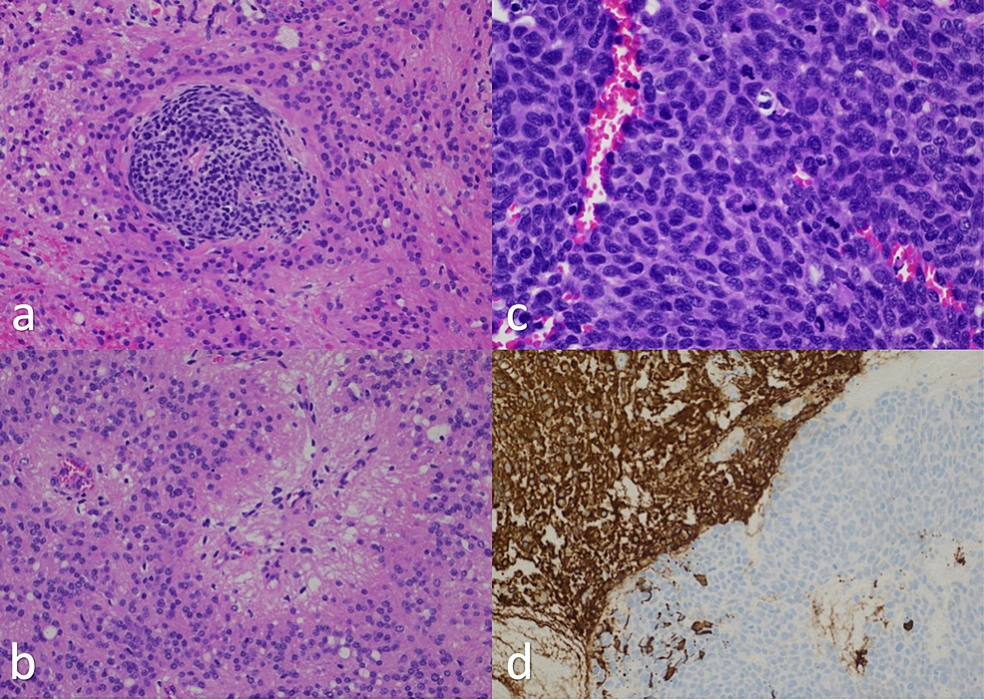
Histopathological features (a) Ependymoma with sarcoma, H&E, 20x. (b) Ependymoma with perivascular pseudo rosettes, H&E, 20x. (c) High-grade sarcoma, H&E, 40x. (d) GFAP stain positive in ependymoma and negative in sarcoma, 20x H&E: hematoxylin and eosin; GFAP: glial fibrillary acid protein

The resection specimen grossly consisted of multiple fragments of tan-pink hemorrhagic friable soft tissue measuring 12.0 x 3.0 x 0.5 cm in aggregate (Figure 5). Microscopically, the tumor consisted of a low-grade ependymoma and a high-grade sarcoma. The ependymoma showed monomorphic cells with round to oval nuclei, salt and pepper chromatin, and perivascular pseudorosettes. The tumor cells were positive for glial fibrillary acid protein (GFAP) and showed a ring-like pattern of the D2-40 expression, endorsing the diagnosis of ependymoma. Ki67 showed a proliferation index of <3%. H3K27me3 expression was maintained in the tumor cells and did not express H3K27M. The retained H3K27me3 is characteristic of the "posterior fossa B" molecular subclass of ependymomas, which occurs more commonly in adults and has a much better prognosis than the "posterior fossa A" subclass that mainly occurs in childhood and manifests the loss of H3K27me3 expression. The sarcomatous component showed malignant spindle cells with nuclear pleomorphism and increased mitoses and necrosis. The spindle cells were negative for GFAP, ruling out a glial neoplasm. Negative staining for S100 and SOX10 along with the absence of the remnants of a schwannoma excluded a malignant peripheral nerve sheath tumor. Ki67 proliferation index was approximately 50%. The final diagnosis was consistent with ependymoma (WHO grade II) and high-grade spindle cell neoplasm of sarcomatous histologic appearance. Next-generation sequencing showed TERT C228T and APC p(Thr1160Lys) mutations. MGMT promoter region methylation assay and next-generation sequencing gene fusion panel were negative.

## Discussion

While sarcomatous changes in gliomas have become increasingly recognized, the limited literature on the topic has mostly focused on astrocytoma and oligodendroglial tumors [6]. Sarcomatous changes in ependymomas [4,7] and subependymomas [8] have received far less attention, ultimately limiting both our clinical diagnoses of these tumors as well as the availability of data on the benefits of surgical treatments for these tumors in a variety of locations. To address this limitation, the current study presented a rare report of a cerebellopontine angle ependymoma that demonstrated rapid progression and sarcomatous change following radiosurgery, and we discuss its clinical management in the context of the current literature.

According to the recent WHO classifications, gliosarcomas are considered a variant of high-grade astrocytoma tumors (GBM) that demonstrates a biphasic mixture of both malignant astrocytic and mesenchymal components [11]. However, for the reasons described above, many have found this definition extremely limited as gliosarcomas can arise from a number of other tumor types outside of high-grade astrocytomas alone [11,12]. As such, the cell of origin for the sarcomatous component of gliosarcomas has long remained a matter of controversy [7]. The previous hypothesis generally claimed that the proliferative blood vessels in the glioma may provide the sarcomatous component of interest through proliferating hyperplastic vascular stroma [10]. While a number of other hypotheses have also been posited, such as those related to endothelial [13] and perithelial origins [14], a monoclonal origin of gliosarcomas has more recently gained interest. This monoclonal origin theory has been supported by numerous cytogenic studies and suggests that the sarcomatous component originates from aberrant mesenchymal differentiation of a malignant glioma or common precursor cell [7,15]. In line with this hypothesis, the sarcomatous component of ependymosarcomas may embody divergent “differentiation” with a new acquisition of mesenchymal features rather than a completely separate neoplasm [7]. While the rationale offered for this “differentiation” remains limited and gliosarcomas generally arise de novo, radiation treatment of an anaplastic neoplasm such as in our patient may induce or accelerate the formation of a sarcomatous component [7]. Therefore, while various forms of differentiation are known to occur in ependymomas, including lipomatous [16], osseous [17], and neuronal [18], it is also necessary to consider ependymal tumors as possible sites of sarcomatous change for early prognostication. Unfortunately, we are limited in this hypothesis due to the fact that no tissue biopsy was obtained prior to radiation in this case. As a result, sarcomatous transformation as a result of radiation therapy remains speculative.

The prevalence of ependymosarcomas will likely continue to increase as anaplastic ependymomas continue to be treated with radiation therapy. The limited descriptions of these tumors in the literature have prevented an effective understanding of their appropriate surgical and post-surgical treatment to date. Many have considered the sarcomatous transformation to be similar to the sarcomatoid changes seen in carcinomas occurring outside the CNS, such as the kidney [19] and prostate [20], and therefore suggested that they likely also represent a more aggressive phenotype. Ultimately, gross total resection may hence provide the most benefit for select patients despite the limited descriptions available [4]. It has been reported that gliosarcomas most commonly occur in the temporal lobe [8]. However, in addition to our case, only one other instance of an ependymosarcoma has been reported to occur in the cerebellopontine angle, and it involved a two-year-old male patient; this was also speculated to occur following radiation therapy and had an overall survival of 25 months following gross total resection [4]. Rodriguez et al., in a diverse case series of 11 ependymosarcomas occurring in a variety of locations, suggested that most patients had poor survival outcomes overall, except for two patients who both underwent gross total resection [4]. The authors concluded that the identification of an ependymal element may carry prognostic significance in select cases.

In this case, given the history of previous radiation, options for re-irradiation were limited. Safe brainstem dosing limits the ability to perform further radiotherapy to the residual tumor at the level of the brainstem. While the use of radiosurgery or proton therapy for re-irradiation was considered in this case, the patient's overall condition declined, leading to the patient and family deciding to cancel any plans for further tumor-directed therapies.

## Conclusions

This report discussed a rare case of an ependymosarcoma occurring in the cerebellopontine angle. Given the history of radiosurgery in our patient, it is presumed that radiation may have induced malignant changes within the lesion. In addition to high-grade astrocytomas, ependymal tumors must also be considered as possible sites of sarcomatous change following radiosurgery. This information may guide future indications for upfront surgical management of lesions with atypical imaging features on diagnosis.
